# Hemodynamic Imaging in Cerebral Diffuse Glioma—Part A: Concept, Differential Diagnosis and Tumor Grading

**DOI:** 10.3390/cancers14061432

**Published:** 2022-03-10

**Authors:** Lelio Guida, Vittorio Stumpo, Jacopo Bellomo, Christiaan Hendrik Bas van Niftrik, Martina Sebök, Moncef Berhouma, Andrea Bink, Michael Weller, Zsolt Kulcsar, Luca Regli, Jorn Fierstra

**Affiliations:** 1 Department of Neurosurgery, University Hospital Zurich, 8091 Zurich, Switzerland; leliog06@gmail.com (L.G.); jacopo.bellomo@usz.ch (J.B.); bas.vanniftrik@usz.ch (C.H.B.v.N.); martina.seboek@usz.ch (M.S.); luca.regli@usz.ch (L.R.); jorn.fierstra@usz.ch (J.F.); 2 Clinical Neuroscience Center, University Hospital Zurich, University of Zurich, 8057 Zurich, Switzerland; andrea.bink@usz.ch (A.B.); michael.weller@usz.ch (M.W.); zsolt.kulcsar@usz.ch (Z.K.); 3 Department of Neurosurgical Oncology and Vascular Neurosurgery, Pierre Wertheimer Neurological and Neurosurgical Hospital, Hospices Civils de Lyon, 69500 Lyon, France; moncef.berhouma@chu-lyon.fr; 4 Department of Neuroradiology, University Hospital Zurich, 8091 Zurich, Switzerland; 5 Department of Neurology, University Hospital Zurich, 8091 Zurich, Switzerland

**Keywords:** hemodynamic, cerebral glioma, glioblastoma, perfusion computed tomography, MRI, perfusion MRI, cerebrovascular reactivity

## Abstract

**Simple Summary:**

Diffuse gliomas, and glioblastomas, in particular, represent a diagnostic and clinical challenge. Standard neuroimaging continues to have many limitations for accurate diagnostic assessment, resection planning and treatment follow-up. The present two-review series comprehensively summarizes recent evidence on hemodynamic imaging applications in the context of diffuse cerebral glioma. Part A provides an overview of the concepts underlying hemodynamic imaging modalities and critically discusses the diffuse glioma differential diagnosis and tumor grading results reported in the literature.

**Abstract:**

Diffuse gliomas are the most common primary malignant intracranial neoplasms. Aside from the challenges pertaining to their treatment—glioblastomas, in particular, have a dismal prognosis and are currently incurable—their pre-operative assessment using standard neuroimaging has several drawbacks, including broad differentials diagnosis, imprecise characterization of tumor subtype and definition of its infiltration in the surrounding brain parenchyma for accurate resection planning. As the pathophysiological alterations of tumor tissue are tightly linked to an aberrant vascularization, advanced hemodynamic imaging, in addition to other innovative approaches, has attracted considerable interest as a means to improve diffuse glioma characterization. In the present part A of our two-review series, the fundamental concepts, techniques and parameters of hemodynamic imaging are discussed in conjunction with their potential role in the differential diagnosis and grading of diffuse gliomas. In particular, recent evidence on dynamic susceptibility contrast, dynamic contrast-enhanced and arterial spin labeling magnetic resonance imaging are reviewed together with perfusion-computed tomography. While these techniques have provided encouraging results in terms of their sensitivity and specificity, the limitations deriving from a lack of standardized acquisition and processing have prevented their widespread clinical adoption, with current efforts aimed at overcoming the existing barriers.

## 1. Introduction

**Figure 1:**
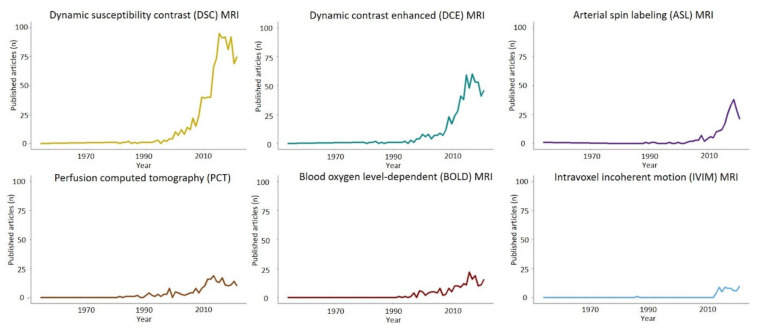
Publication per years of different hemodynamic imaging modalities and gliomas. DSC and DCE-MRI have been the subject of more intense research in glioma imaging, followed by ASL-MRI and PCT. IVIM-DWI and BOLD-CVR can also provide different hemodynamic information and in recent years are becoming the focus of active research.

Cerebral diffuse gliomas are the most malignant primary and frequently diagnosed intracranial tumors [1,2]. The most common entity is represented by glioblastoma, an incurable brain tumor whose standard of care since the seminal trial by Stupp et al. has included temozolomide (TMZ) on top of radiotherapy (RT) following a maximally safe surgical resection [2,3]. Despite their low prevalence, these brain tumors are associated with a dismal prognosis, incommensurable emotional and social burden and high treatment costs [4,5]. The standard imaging protocol for suspected diffuse adult glioma evaluation includes different sequences of magnetic resonance imaging (MRI), such as pre- and post-gadolinium contrast-enhanced (CE-) T1-weighted imaging, T2-weighted sequences including Fluid Attenuated Inversion Recovery (FLAIR) imaging, which are often complemented by diffusion-weighted imaging, susceptibility-weighted sequences and perfusion-weighted imaging (PWI) for a more refined diagnostic imaging workup [2,6]. The drawbacks of morphological MRI sequences are well-recognized and result in a broader differential diagnosis because other lesions, such as cerebral lymphoma, metastasis and abscesses, can have a similar radiological presentation during standard neuroimaging [7]. Additional limitations include imprecise characterization of glioma grading and subtype allocation for treatment decisions [8], as well as suboptimal determination of the extent of tumor infiltration for accurate resection planning [9]. Furthermore, challenges occur when differentiating glioma progression/recurrence from treatment-related effects such as radiation necrosis, pseudo-progression and pseudoresponse [10,11,12,13], as well as imprecise prognostication [14], whereby the different molecular alterations that are becoming more sophisticated and clinically relevant to define the disease entity can hardly be derived from the pre-operative MRI [15]. Of note, the WHO classification 2021 further defines adult diffuse gliomas based on a combined clinical and histological grading, whereby, for example, glioblastoma can be diagnosed only in the presence of IDH wild-type status (eliminating IDH mutant glioblastoma), and astrocytoma, IDH mutant, now also includes a grade 4 variant [15]. Importantly, to date, histopathological diagnosis confirmation and molecular characterization remain the gold standard [15], but advanced imaging developments over the last three decades have fostered a variety of hemodynamic imaging investigations with the potential to provide complementary information regarding tumor type, aggressiveness and molecular correlates [16,17,18] (Figure 1).

These techniques and others have all been exploited to foster progress in pre-operative diffuse glioma assessment and follow-up, with the use of various multimodality approaches becoming the new standard of care [6,9,19]. Hemodynamic glioma imaging is a growing topic where the abundance of technical imaging advancements and the ever-growing literature can be hard to decipher and contextualize [20]. For this reason, we present the current review as a two-part investigation where we aim to provide an updated overview of the different hemodynamic imaging modalities for diffuse glioma assessment. In the current manuscript (i.e., Part A of the review), the fundamental concepts of hemodynamic imaging are discussed in conjunction with their potential role in diagnosis and grading gliomas. Of note, hemodynamic imaging has historically been investigated extensively to differentiate between higher-grade and lower-grade glioma subtypes based on previous WHO classifications [21,22]. For the present review, the now-outdated high-grade glioma definition (i.e., including astrocytoma IDH-mut grade 3, as well as IDHwt and IDHmut glioblastoma) is used to discuss the previous findings. For this reason, we caution the reader to interpret this evidence while maintaining the most recent WHO CNS classification in mind [15]. Part B is a consecutive separate review where we discuss the potential use of hemodynamic imaging techniques to distinguish post-treatment effects, i.e., pseudoprogression and radiation necrosis from true tumor progression or recurrence, as well as diffuse glioma molecular characterization and prognostication, together with the latest advancements in new techniques, the integration of radiomics and the computational advantage granted by machine learning methods.

## 2. Overview of the Techniques and Parameters

In normal physiology, cerebral vessel regulation and brain hemodynamics are highly sophisticated and fine-tuned by changes in blood gases, neuronal and tissue metabolism and blood pressure [23], and it is thus not unexpected that in diffuse gliomas, the pathophysiological and histological alterations determined by the growing tumorous tissue heavily affects these processes and results in abnormal hemodynamics. The determinants of such changes are several and only partly understood. Firstly, the highly heterogeneous growing tumor tissue displays high and deranged metabolic activity [24,25] at its initial stages, partially sustained by the subversion of microvascular anatomy, including extensive vascular remodeling and tumor angiogenesis controlled through the hypoxia-induced expression of vascular endothelial growth factor (VEGF). This results in irregular, disorganized and tortuous vessels with arteriovenous shunting and increased permeability of the defective BBB [26,27], accompanied by the spreading of tumor cells in the perivascular spaces—or vessel co-option [27,28,29,30,31]. At later stages, disruption of the blood–brain barrier, protein extravasation, hemorrhages and extensive necrosis histologically characterized by pseudopalisades occur [29,32]. The normally tight coupling between neuronal activity and hemodynamic changes in nearby vessels that are the target of functional MRIs for task-related presurgical mapping is also affected by the brain tumor, i.e., neurovascular uncoupling (NVU) resulting in a false-negative activation [33]. Furthermore, vasogenic and cytotoxic brain edema surrounding the lesion also impacts intracranial pressure, with consequences on the cerebral hemodynamic (e.g., decreased cerebral blood flow, impaired cerebrovascular reactivity, etc.) [34,35,36]. The ensemble of these alterations makes hemodynamic imaging particularly attractive in investigating pathophysiological alterations in gliomas. Hemodynamic brain imaging can be conceptually divided into the imaging of perfusion and that of cerebrovascular reactivity (CVR). The former is based on techniques studying the passage of blood through the vasculature and its relationship with the blood–brain barrier (BBB) and extravascular space. The latter focuses on analyzing the reactivity of the cerebral vessels and subsequent flow redistribution.

### 2.1. Perfusion Imaging

Perfusion imaging follows the blood in the vascular system up to the tissue of interest using indicators (or tracers). These can be divided into two classes: intravascular, which remain in the vessels in physiological conditions, and freely diffusible, which can leave the intravascular space and diffuse throughout the entire tissue volume [37,38]. The measurement of perfusion using intravascular tracers is based on the indicator dilution theory, first described using the Meier–Zierler model [39], whereas the use of freely diffusible ones relies on Fick’s diffusion law and was first described by the Kety–Schmidt model [40]. The perfusion imaging of brain tumors adopts several techniques, including dynamic susceptibility contrast (DSC)-MRI, dynamic contrast-enhanced (mit)-MRI, arterial spin labeling (ASL)-MRI [20] and perfusion computed tomography (PCT). Positron emission tomography (PET) and single-photon emission computed tomography (SPECT) can also be used to assess brain perfusion [41,42], but their use in brain tumor assessment has been seldom reported. Altogether, these techniques are based on different methodological approaches, present specific limitations and allow for the assessment of different perfusion parameters in the study of gliomas [18,41,42,43,44,45,46,47,48,49,50] (Table 1). These measures correlate to the pathophysiological underlying features of the studied tissue. For example, tumor cerebral blood volume (CBV) represents a good surrogate marker for microvascular density [51,52], a measure of angiogenesis that is an important prognostic indicator in malignant brain tumors [53,54,55], while the permeability parameters are specifically suited to assessing in vivo BBB leakiness present in the dysfunctional glioma vasculature [51] (Table 2). 

**Figure 2:**
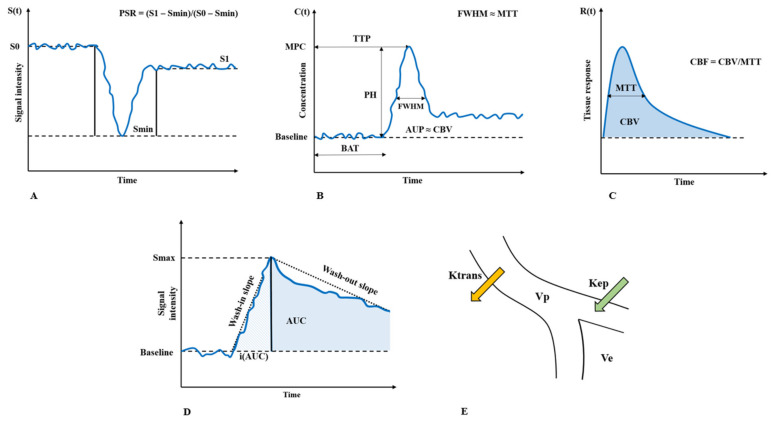
The derivation of perfusion parameters from the signal-response time curve is shown. The signal response time-curve is acquired during contrast bolus passage in the studied region-of-interest. From the signal response time-curve the changes of bolus concentration are estimated (tissue bolus concentration time-curve). Tissue bolus concentration time-curve is processed with mathematical models enabling a qualitative, semi-quantitative or quantitative assessment/measurement of perfusion parameters. Panel (**A**) Simplified signal response time curve acquired during DSC-MRI. Panel (**B**) Simplified tissue bolus concentration time-curve. Panel (**C**) Deconvoluted tissue bolus concentration time-curve to tissue response time-curve. Panel (**D**) Simplified signal response time-curve acquired during DCE-MRI. Panel (**E**) Schematic representation of permeability parameters derived from DCE-MRI. (Adapted from Zhang, J.; Liu, H.; Tong, H.; Wang, S.; Yang, Y.; Liu, G.; Zhang, W. Clinical Applications of Contrast-Enhanced Perfusion MRI Techniques in Gliomas: Recent Advances and Current Challenges. *Contrast Media Mol. Imaging* **2017**, *2017*, 7064120. https://doi.org/10.1155/2017/7064120). Abbreviations: PH, peak height; PSR, percentage signal recovery; other abbreviations are defined in Table 2.

DSC-MRI, DCE-MRI and PCT are the available CE perfusion imaging techniques [37,56], and they all share the same principle regardless of the underlying analysis model: the labeling of circulating blood with a bolus injection of a contrast agent and the tracking of dynamic imaging signal changes consequent to the first pass of labeled blood in the observed region of interest (bolus-tracking method) [37,39,56]. Of note, the absolute quantification of the derived perfusion parameters is possible only if the arterial input function (AIF) is accounted for in the data analysis, i.e., the deconvolution processing of the tissue bolus concentration time-curve [57,58]. AIF describes the time-dependent bolus concentration input curve to the tissue and it is required to account for the confounding effects resulting from bolus dispersion antecedent to its arrival in the region of interest (ROI) [57]. To determine AIF, several methods are known (refer to Calamante et al. for more details) [57]. As the measurement of the AIF is challenging in the perfusion imaging of brain tumors, these parameters are often directly derived from the tissue bolus concentration time-curve normalized to an area of “normal” tissue and therefore reported in a semi-quantitative form [58,59] using the contralateral white matter as the preferred reference [60] (Figure 2).

In this respect, it is important to note that in the literature, the term “relative” (e.g., rCBV) is usually used to define semiquantitative parameters but the term “normalized” (e.g., nCBV) can be used interchangeably. Confusion can arise between the terms “relative” and “regional”, with the second referring to absolute measurements. In the present work, unless otherwise specified, we refer to the relative semiquantitative parameters and the r/n prefix has been omitted to enhance readability.

### 2.2. Cerebrovascular Reactivity Imaging

**Figure 3:**
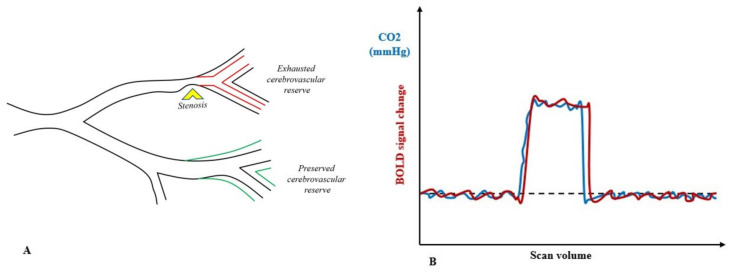
Cerebrosvascular reactivity. Panel (**A**) shows a schematic of encroached vasodilatory reserve and downstream of stenosis. Upon vasodilatory stimulus, all vessels will be stimulated to dilate, but flow increase in those with preserved vasodilatory reserve will reduce the flow distal to regional resistance. (Adapted by Sobczyk, O.; Battisti-Charbonney, A.; Fierstra, J.; Mandell, D.M.; Poublanc, J.; Crawley, A.P.; Mikulis, D.J.; Duffin, J.; Fisher, J.A. A conceptual model for CO2-induced redistribution of cerebral blood flow with experimental confirmation using BOLD MRI. *NeuroImage* **2014**, *92*, 56–68. ISSN 1053-8119. https://doi.org/10.1016/j.neuroimage.2014.01.051). Panel (**B**) shows a controlled standardized hypercapnic stimulus and its correlation to BOLD signal change.

Two mechanisms govern the cerebrovascular system by controlling the vascular smooth muscle cell tones to help regulate the regional CBF: autoregulation, which ensures the maintenance of blood flow in response to changes in perfusion pressure [68], and metabolic neurovascular coupling, which increases the blood flow in active brain areas [69]. Cerebrovascular reactivity is a hemodynamic parameter describing the ability of brain vessels to obey these regulatory mechanisms. It is measured through the application of a vasoactive (vasodilatory or vasoconstrictive) stimulus and defined as the change in cerebral blood flow (CBF) per change in the given stimulus [70]. Among the different vasodilatory stimuli (such as hypotension, acetazolamide, carbon dioxide) that have been described, carbon dioxide is the most attractive due to its many advantages [70]. Blood oxygen level-dependent (BOLD) functional MRI (fMRI) with a carbon dioxide respiratory challenge belongs to calibrated fMRI techniques [71] and is a reliable, accurate and reproducible imaging technique to assess cerebrovascular reactivity [66,70,72] (Figure 3).

With the introduction of end-tidal targeting gas delivery systems, the possibility granted by precise independent control of the end-tidal pressure of carbon dioxide and oxygen [73] has increased the intra- and inter-subject reproducibility of the technique [65,73]. In recent years, the study of CVR has also proved a valuable adjunct for glioma characterization [74,75,76].

## 3. Clinical Applications of Hemodynamic Imaging in Cerebral Diffuse Gliomas—Part 1

After having reviewed the basic principles of hemodynamic imaging, we now present how their application has been investigated in the clinical work-up of patients with high-grade gliomas and discuss the relevant differences in the assessed parameters among the different tumor entities.

### 3.1. Differential Diagnosis versus Other Neoplastic and Non-Neoplastic Lesions

The first magnetic resonance presentation of a brain lesion showing a heterogeneously enhancing mass surrounded by extensive peritumoral edema on a T2-weighted image is not univocally interpretable. Hemodynamic imaging has been investigated with the aim of differentiating between gliomas and other possible neoplastic entities such as brain metastases (BM) [77,78,79,80,81,82,83,84,85,86,87,88,89,90,91,92,93,94,95,96,97,98,99,100] and cerebral lymphoma [77,78,81,82,84,85,88,89,91,92,96,97,98,100,101,102,103,104,105,106,107,108], as well as other lesions like abscesses or demyelination [87,109,110,111].

#### 3.1.1. Metastases

In an ideal case scenario, metastases should always be distinguished pre-operatively from high-grade gliomas to ensure a complete patient diagnostic assessment and to optimize the treatment decision-making [112]. BM, differently from invasive high-grade gliomas, are encapsulated lesions. This renders the hemodynamic study of peritumoral tissue particularly suited for a possible differentiation of the two entities (vasogenic versus infiltrated edema) [113,114]. DSC-MRI studies have provided the most consistent results on discriminating between high-grade gliomas and metastases [77,83,95,96,97,98,99,115,116]. In fact, a higher DSC-derived CBV can be found in high-grade glioma peritumoral edema [83,95,99]. Measuring the peritumoral CBV possesses the additional advantage of avoiding possible confounding due to hypervascular metastasis, e.g., melanoma, a diagnostic issue also reported in DCE studies [17,80,115]. In their meta-analysis including 900 patients from 18 studies, Suh et al. found that the use of DSC-derived peritumoral CBV provided the best results in terms of sensitivity (0.89) and specificity (0.88), with an AUC of 0.96 [115]. Because of the heterogeneity in the scan parameters and data processing, the cut-off values reported varied. Differently from CBV, PSR is thought to reflect a combination of many factors including CBF, the volume of extravascular space and contrast leak. This parameter has also been reported to be higher in high-grade gliomas compared to metastases [77,97,117], probably due to the different histological structures in metastatic tissue with respect to high-grade gliomas, with the former displaying endothelial gaps and an absence of BBB—determining the loss of signal recovery—and the latter only displaying a decreased capillary fenestration and partial BBB disruption. Other studies have found there to be no difference between the two entities [96,116]. Even if less investigated, DCE-MRI also showed potential for high-grade glioma versus metastasis differentiation [80,82,94,95], despite some reports failing to find relevant significant differences [95,118]. Although the same meta-analysis by Suh et al. included only two studies using DCE, the authors still highlight how this technique can constitute a better alternative given the drawback of DSC susceptibility to surgery-dependent artifacts. DCE-derived iAUC and Ktrans were found to be higher in the peritumoral white matter of glioblastomas compared to metastases [94]. Previously, Jung et al. reported that pharmacokinetic parameters, such as lesional Ktrans and Vp, cannot differentiate between the two tumor entities, while hypovascular metastases can be differentiated by the AUC signal-intensity curve and wash-out log slope from glioblastoma and melanoma (hypervascular) metastases [80]. In a small series, Bauer et al. found that, despite the Ktrans being higher in non-enhancing T2 hyperintensities of glioblastoma, this did not reach statistical significance [95]. Contrary to the other observations, Zhao et al. in 2015 reported Ve and iAUC to be higher in metastases than in high-grade gliomas. However, this study only included five metastases patients—all from the hypervascular primaries [82]. These findings stress how, in addition to the variability of acquisition and processing methodology, the histology of the primary tumor has a role in perfusion assessment and thus needs to be carefully evaluated in future studies. In line with the findings from DSC, Lin et al. found that high-grade gliomas display higher absolute and relative ASL-derived CBF than metastases in peritumoral edema, contrarily to the absence of differences in enhancing lesions [79]. Other series instead found CBF to also be higher intratumorally [93,108,119]. Despite fewer studies, ASL is also included in the meta-analysis by Suh et al. The authors concluded that this technique also exhibits good potential for differentiation [120]. In this respect, in 2019, Fu et al. conducted a meta-analysis to assess ASL performance. They included five studies with a total of 346 patients and concluded that ASL has high sensitivity, specificity and AUC for differentiating brain metastases from gliomas [121]. PCT studies have also found there to be decreased CBV and MTT in metastases compared to gliomas [78], while a recent series by Onishi et al. reported that PCT-derived peritumoral MTT (higher in metastases) and CBF achieved the best performance for differentiating high-grade gliomas from other intracranial tumors, with CBV also showing statistical differences [100]. Despite some encouraging findings, the conflicting evidence remains to be evaluated and it should be noted that the most recent EANO guidelines on brain metastases state that perfusion MRI does not accurately discriminate BM from malignant tumors of glial origin [112].

#### 3.1.2. Primary Central Nervous System Lymphomas

The distinction between gliomas and cerebral lymphomas is even more sensible, as the latter should be treated with chemoradiotherapy with the role of resection remaining controversial [122,123]. DSC-MRI studies found that, with respect to high-grade gliomas, cerebral lymphomas show lower CBV [77,91,92,96,103,105,106,124]. This is in line with absent angiogenesis in these tumors—lower CBF [124], higher PSR [77,96,97,124] (characteristically displaying a phenomenon of overshooting, not fully understood but possibly determined by dominant T1 effects of extravasated contrast agent accumulating in the interstitial space [77]), lower PH [96] and lower MTT [124]. A meta-analysis by Xu et al. found that DSC-derived measures are optimal for distinguishing high-grade gliomas from cerebral lymphomas (AUC: 0.98; sensitivity 0.96) [125]. DCE studies found increased Krans [102,106,118], increased Kep [107], increased Ve [102,118,126], lower Vp [127] and increased iAUC [82,128] versus high-grade gliomas. Conflicting evidence was reported by Kickingereder et al., who did not find increased Ve [107], and Lin et al., who failed to report a significant difference in Ktrans [127]. Taken together, the results regarding the permeability parameters show an increased leakiness through BBB in cerebral lymphomas that have been associated histologically with tumor invasion of the basement membrane, as well as the absence of/thinner endothelial cells as opposed to high-grade gliomas [102,107]. iAUC’s underlying pathophysiological correlates are still debated, but as a model-free metric (see Table 2), it likely still reflects a combination of different perfusion variables [128]. Due to the lower amount of evidence, the meta-analysis by Suh et al. concluded that robust conclusions on DCE could not be drawn due to the lack of enough evidence [120]. Okuchi et al. in 2019 published another meta-analysis including five DCE studies (224 patients) and reported a pooled sensitivity and specificity of 0.78 and 0.81, with an AUC of 0.86 [129]. ASL studies found a decreased CBF in cerebral lymphomas versus high-grade gliomas likely resulting from absent angiogenesis in these lesions [102,108,130]. Suh et al. also ran a meta-analysis to assess the diagnostic performance of perfusion MRI in differentiating glioma from cerebral lymphomas. They found that ASL studies, the same as DSC, showed a high diagnostic performance with a sensitivity of 0.93 and a specificity of 0.91 [120]. Another meta-analysis by Xu et al. found that ASL had the best specificity, i.e., 0.90, for this purpose when compared with DSC and DCE. The variability of the reported threshold calls for further standardization, which is currently lacking [125]. PCT studies found decreased CBF [100,104] and CBV [78,85,100,104,104], lower MTT [78] and higher Ktrans [104,131] (not found by Schramm et al. [85]). For cerebral lymphomas, despite the EANO guidelines recognizing the role of perfusion MRI in differentiating between them (DSC-MRI in particular), histopathological confirmation is required for diagnosis as no imaging modality possesses a sufficient specificity [122].

#### 3.1.3. Non-Neoplastic Lesions: Abscesses and Autoimmune Lesions

Furthermore, cerebral abscesses can also sometimes be difficult to distinguish from high-grade gliomas as these also present as a CE rim surrounding a central necrotic core, with diffusion-weighted imaging being particularly useful for this purpose [132,133]. Perfusion imaging can provide an additional tool to better differentiate between these two lesions, despite only a handful of small series and case reports having addressed this topic in the published literature. In particular, differences in the contrast-enhancing rim have attracted interest. Toh et al. reported the CBV in the enhancing rim of abscesses to be significantly lower than those in GBM or metastases [109], in agreement with previous studies [87,88,132,134,135]. Hakyemez et al. also reported the CBF to be increased in high-grade gliomas, with respect to infectious lesions where Ktrans and Ve are reduced [88]. Regarding PCT, a recent study by Karegowda failed to find any significant difference between high-grade gliomas and abscesses [78]. This is different from a previous study by Chawalaprit et al. [136]. Similar to infectious lesions, tumefactive demyelinating lesions (TDLs) have also been investigated in small series with conflicting results. In fact, despite the highly angiogenic tumor tissue having different histopathological alterations, some of these lesions can mimic high-grade gliomas in standard imaging leading to unnecessary treatment. DSC-derived CBV failed to differentiate TDLs from WHO grade 2 and grade 3 gliomas in a retrospective series by Blasel et al. [137], whose low sensitivity was also confirmed by Hiremath et al. [138]. Other series found lower CBV [139,140] and CBF [140] in the former. A study by Jain et al. assessing PCT also reported CBV, CBF and PS to be lower in high-grade gliomas compared to TDLs [110].

### 3.2. Glioma Grading/Subtype

The standard imaging of gliomas can lead to an educated guess on the expected tumor grade, but as some low-grade gliomas can present with contrast-enhancement and a number of atypical high-grade gliomas do not readily exhibit BBB disruption, hemodynamic imaging can further support the differentiation of these two entities. Low-grade gliomas are mostly characterized by native vessel co-option while the hypoxic angiogenic microenvironment of high-grade gliomas presents with an increased number of leaky vessels [141]. These features are reflected by an increased microvascular proliferation in more aggressive tumors (leading to increased CBV and CBF) and an increase in the permeability parameters describing BBB leakiness. Numerous publications have assessed the performance of hemodynamic imaging in glioma grading, in particular when distinguishing high-grade from low-grade gliomas [8,77,78,81,82,84,86,104,105,111,142,143,144,145,146,147,148,149,150,151,152,153,154,155,156,157,158,159,160,161,162,163,164,165,166,167,168,169,170,171,172,173,174,175,176,177,178,179,180,181,182,183,184,185,186,187,188] but also when distinguishing grade 3 from grade 4 [148,153,155,162,169,173,178,182,189,190,191]. It is important to note once more that all of these studies investigated imaging performance related to the previous WHO classification [21,22]. The changes implemented in the latest classification [15] deserve there to be a contextualization of the past findings of hemodynamic imaging in the framework of the newly available evidence. In general, while the differentiation of high-grade versus low-grade gliomas has been more strongly established, the extent to which these techniques can distinguish between grade 3 and grade 4 gliomas has reported more conflicting results. This is where the authors inconstantly report significant differences in some parameters or the failure of perfusion imaging to differentiate them [153,162,173,178,192]. Most notably, DSC-CBV has been consistently found to be higher in high-grade gliomas [8,77,88,98,142,146,148,151,158,160,166,170,174,179,180,181,182,187,188,191,193,194,195,196] with occasional reports finding no difference [150]. These associations have been suggested to be useful when seeking to identify possible biopsy sampling errors, e.g., cases where the histopathology and CBV are discordant [197,198]. Of note, a recent study by Gaudino et al. reported that CBV has a different optimal threshold for tumor grading in supratentorial versus infratentorial tumors [98]. The overall consistency of the reported findings was confirmed by recent meta-analyses [199,200]. A recent study pooled the quantitative CBV values through a random-effect meta-analysis confirming that these were higher in high-grade than low-grade gliomas. In particular, DSC-CBV had a pooled sensitivity of 0.92 and a pooled specificity of 0.81 with a pooled area of 0.91 under the curve (AUC) [199]. In another meta-analysis, it was investigated whether DSC can differentiate between grade 2 and grade 3 gliomas. It was accordingly reported that the latter had a higher CBV [200]. Nevertheless, a Cochrane Review by Abrigo et al. published in 2018 used the DSC-CBV threshold of <1.75 to differentiate low-grade gliomas from non-enhancing high-grade gliomas and found that the summary of the sensitivity/specificity estimates was 0.83/0.48, with specificity rising to 0.67 when using only five good-quality studies in the sensitivity analyses. For this reason, the authors concluded that DSC cannot yet reliably be used for this purpose due to possible detrimental repercussions on the treatment strategy approach [201]. DSC-CBF has also been reported to be higher in high-grade gliomas [8,88,160,170,171,177,179,187,193,194,202] with occasional studies reporting no significant difference in this parameter [175]. Even if less commonly investigated, other parameters that can be derived from DSC-MRI are correlated to glioma grade. For example, MTT has been reported to be higher in high-grade gliomas [8] but surprisingly, Alkenhal et al. found it to be lower in non-enhancing grade 3 vs. grade 2 [150], while some reports found there to be no difference between high-grade and low-grade gliomas [179]. PH and TTP (in peritumoral edema) are significantly lower and higher respectively, in high-grade as compared with low-grade gliomas [8,182]. PSR has been reported to be decreased in high-grade gliomas [170,174], with some reports failing to find a difference [77]. DCE-MRI studies consistently reported increased Ktrans [82,152,166,173,183,203,204,205,206,207], which constitutes the most commonly investigated parameter. Despite this indicator in healthy conditions primarily reflecting BBB permeability, it should be treated with caution in cases of disrupted BBB—such as in high-grade gliomas—where it is more reflective of and limited by blood flow (see Table 2) [208]. Other DCE-derived metrics that are increased in high-grade gliomas include Ve [82,182,203,204,205,206], Vp [166,173,209] and AUC [82]. Despite these congruent findings, other authors, most likely because of methodological differences, insufficient sample sizes or different statistical analyses, failed to find significant differences in Ktrans [210], Ve [203] and Kep [203] between high- and low-grade lesions. Okuchi et al. meta-analyzed 14 DCE studies and found that the pooled sensitivity, specificity and AUC for differentiating high-grade from low-grade gliomas were 0.93, 0.90 and 0.96 [129]. ASL-derived CBF has also been consistently reported to be higher in high-grade gliomas [145,147,153,154,156,160,163,164,167,168,171,172,177,211] with sporadic reports failing to show a difference [183]. Some studies have also compared the CBF derived from ASL to DSC-CBF and DCE-CBF. It is relevant to note that one such study by Roy et al. reported a poor correlation between DCE and ASL for CBF calculation [183], while Hashido et al. found in their study that ASL underestimates CBF with respect to DSC [147]. Several meta-analyses conducted to assess the role of ASL in glioma grading concluded that ASL possesses a good discriminative ability for glioma grading [199,212,213,214]. Kong et al. performed a meta-analysis using a random effect model and included 9 studies for a total of 205 patients, reporting that both the relative and absolute TBF values were significantly increased in the high-grade gliomas with respect to the low-grade gliomas [212]. One year later, Delgado et al. conducted a systematic review and analyzed 15 studies including 505 patients and concluded that both pseudocontinuous and pulsed ASL-calculated CBF possess excellent diagnostic performance in terms of glioma grading (AUC 0.90 and 0.88, respectively) [213]. A recent study pooled quantitative CBF data into a random-effect meta-analysis confirming that they were more high-grade gliomas with a pooled sensitivity of 0.88 and a pooled specificity of 0.91. In the same investigation, the calculated pooled AUC was 0.95 [199]. Another systematic review by Alsaedi et al. found that absolute CBF can differentiate low-grade from high-grade gliomas but it loses its discriminative ability when only grade 2 versus grade 3 or grade 3 versus grade 4 gliomas are compared [214]. The authors warn that because of the different ASL approaches (pulsed-ASL, pseudo-continuous ASL and continuous ASL) and acquisition parameters, the quantitative measurements reported in the single studies are to be interpreted with caution. Moreover, other shortcomings of the different methodologies applied are critically discussed, such as poor labelling efficacy and unstandardized ROI choice. The PCT series has also reported that using this technique can be used to differentiate high-grade from low-grade gliomas with CBV, as CBF was higher in the former [78,215] while no differences in MTT [78,215] and TTP were found [78]. In addition to increased CBV, PCT also reported increased PS in high-grade gliomas [216].

## 4. Limitations

To overcome the limitations intrinsic to the various imaging modalities (e.g., CBV measurement by DSC is influenced by GRE vs. SE pulse sequence, contrast agent dosing, image acquisition parameters, post-processing techniques and GBCA leakage correction), efforts were made to achieve protocol standardization aimed at solving the variability of the reported methodologies and their resulting reported cut-offs [217,218,219]. Similar consensus papers aimed at solving the issues associated with ASL acquisition [220] and, more importantly, the processing pipeline are also underway [221].

## 5. Conclusions

Hemodynamic imaging-derived measurements are well suited to characterizing abnormal vasculature perfusion, BBB defects and providing complementary information with respect to standard neuroimaging to assist in diffuse cerebral glioma patient diagnostic workup. Several studies have assessed the potential of hemodynamic imaging for brain tumor differential diagnosis and tumor grading. They reported high sensitivity and specificity, especially of DSC but also of DCE and ASL-MRI for the differentiation of high-grade gliomas from brain metastases, cerebral lymphomas and non-neoplastic lesions, as well as for differentiating high-grade gliomas from low-grade gliomas. As recent efforts aim to tackle the standardization issues intrinsic to the imaging modalities investigated (from hardware and acquisition parameters to model analysis and post-processing), a number of limitations have prevented the univocal interpretation of the previous findings, largely limiting a robust and widespread clinical translation.

## Figures and Tables

**Table 1:** Overview of hemodynamic imaging techniques. Abbreviations: AIF, arterial input function; ASL, arterial spin labeling; BBB, blood–brain barrier; BOLD, blood-oxygen-level-dependent; CA, contrast agent; CVR, cerebrovascular reactivity; DSC, dynamic susceptibility contrast; DCE, dynamic contrast-enhanced; GBCA, gadolinium-based contrast agent; IBCA, iodine-based contrast; MTT, mean transit time; PCT, perfusion computed tomography; PET, positron emission tomography; SPECT, single-photon emission computed tomography.

	DSC-MRI	DCE-MRI	ASL-MRI	BOLD-CVR	PCT	PET	SPECT
Contrast agent	GBCA	GBCA	-	-	IBCA	15-O2, H2150, C15O2	133Xe, 99mTc-HMPAO, 99mTc-ECD, 123i-IMP
Radiation exposure	-	-	-	-	+++	+	-
Data model analysis	Meier–Zierler [39]	Meier-Zierler [39]; Tissue-homogeneity model, modified Tofts model, three-parameter models, two-parameter models, on-parameter models [47]	Kety–Schmidt [40]	Fürst et al. [61]	Meier–Zierler [39]	Kety–Schmidt [40]	Kety–Schmidt [40]
Assessed parameters *	CBV, CBF, MTT	Ktrans, Ve, Vp, Kep (CBV, CBF, MTT)	CBF	CVR	CBV, CBF, MTT, Ktrans, Ve, Vp, Kep	CBF, CBV, OEF	CBF
Strenghts	Lack of radiation exposure and use of iodinated CA; Combination with standard MRI sequences for a more comprehensive assessment of brain tumors	Lack of radiation exposure and use of iodinated CA; Combination with standard MRI sequences for a more comprehensive assessment of brain tumors; Higher spatial resolution than DSC	Non-invasive No need of GBCA	Non-invasive No need of GBCA	Linear relationship of tissue signal intensity with tissue contrast agent, allows measurement of permeability parameters	Accurate quantitative measurements Repeatibility due to short half of radiotracers	Low costs, Feasibility in emergency settings
Limitations	Indirect detection of the injected CA; Competing T1 contrast effect due to CA leakage through BBB **; Challenging measurement of AIF	Indirect detection of the injected CA; Choice of the most appropriate analysis models among the different existing ones; High temporal resolution required; Dependency from the CA extraction fraction Challenging measurement of AIF	Poor labeling efficiency, blood transport through vessels and tissue, proton water diffusion through the BBB, low SNR, high sensitivity from patient motion and magnetization transfer effects. Challenging measurement of AIF	Possible light patient discomfort due to carbon dioxide stimulus	Reduced anatomic coverage	High costs, impossibility to use in the emergency clinical settings	Poor spatial resolution
Suggested readings	Shiroishi et al. [62] and Quarles et al. [45]	Sourbron and Buckley [47]	Buxton et al. [63] and Calamante et al. [37]	Buxton et al. [64] Fisher et al. [65,66]	Jain et al. [51]	Zhang et al. [67]	Zhang et al. [67]

* For definition of assessed parameters see Table 2. ** These leakage effects can be reduced by the commonly used preload leakage-correction strategy and by applying different model-based leakage-correction algorithms. (Qaurles et al., Bjorneurd et al., Boxerman et al., Donahue et al., Leu et al.).

**Table 2:** Perfusion parameters. Abbreviations: BBB, blood-brain barrier; CA, contrast agent; EES, extravascular extracellular space; g, grams; min, minute; mL, milliliter; ROI, region of interest; s, second.

Parameter	Interpretation	Explanation	Units
CBV	Cerebral blood volume	Quantity of blood in a given amount of brain tissue. It is considered a surrogate of microvascular density.	mL of blood/100 g tissue
CBF	Cerebral blood flow	Rate of delivery of arterial blood to a capillary bed in tissue.	mL of blood/100 g of tissue/min
MTT	Mean transit time	Average time that red blood cells spend within a determinate volume of capillary circulation. It is calculated as CBV/CBF.	s
Ktrans	Volume transfer constant between blood plasma and extravascular extracellular space	Measure of capillary permeability, is considered a good indicator of BBB leakiness. It should be noted that in situation of high permeability (disrupted BBB) this parameter is more reflective of CBF.	1/min
Ve	Extravascular extracellular volume fraction	Quantification of cellularity and necrosis in extravascular extracellular space	mL/100 mL
Vp	Blood plasma fractional volume	Quantification of the volume of blood plasma	mL/100 mL
Kep	Rate constant from extravascular extracellular space into blood plasma	Flux rate constant between the EES and blood plasma. It can be derived as Ktrans/Ve.	1/min
TTP *	Time to peak	Time at which contrast concentration reaches its maximum.	s
BAT *	Bolus arrival time	Time from CA bolus injection to measured concentration changes in the observed ROI	s
MPC *	Maximum peak-concentration	Maximal CA concentration in the observed ROI	mL/100 mL
FMWH *	Full-width at half-maximum concentration	Measure of the width at half the maximum value of peaked concentration–time curve	s
AUP *	Area under the peak	Area under the peaked concentration–time curve	-

* Summary parameters. These are directly quantified by measuring summary properties of the tissue bolus concentration time-curve (“curvology”), and are therefore model-free metrics that do not possess specific physiological foundations and most likely represent a combination of different hemodynamic parameters (e.g., CBV, CBF, vessel permeability) and technical aspects (e.g., imaging technique, contrast dose, injection rate).
